# Fear of COVID-19, Anxiety and Depression in Patients with Pulmonary Arterial Hypertension and Chronic Thromboembolic Pulmonary Hypertension during the Pandemic

**DOI:** 10.3390/jcm10184195

**Published:** 2021-09-16

**Authors:** Maria Wieteska-Miłek, Sebastian Szmit, Michał Florczyk, Beata Kuśmierczyk-Droszcz, Robert Ryczek, Milena Dzienisiewicz, Adam Torbicki, Marcin Kurzyna

**Affiliations:** 1 Centre of Postgraduate Medical Education, European Health Centre, Department of Pulmonary Circulation, Thromboembolic Diseases and Cardiology, ul. Borowa 14/18, 05-400 Otwock, Poland; s.szmit@gmail.com (S.S.); michal_florczyk@wp.pl (M.F.); adam.torbicki@ecz-otwock.pl (A.T.); marcin.kurzyna@ecz-otwock.pl (M.K.); 2 Department of Congenital Heart Disease, National Institute of Cardiology, ul. Alpejska 42, 04-628 Warsaw, Poland; bkusmier@gmail.com; 3 Department of Cardiology and Internal Diseases, Military Institute of Medicine, ul. Szaserów 128, 04-349 Warsaw, Poland; raryczek@gmail.com; 4 European Health Centre, 05-400 Otwock, Poland; milena.dzienisiewicz@gmail.com

**Keywords:** fear of COVID-19, pandemic, anxiety, depression, HADS scale, pulmonary hypertension, pulmonary arterial hypertension, chronic thromboembolic pulmonary hypertension

## Abstract

The COVID-19 pandemic has affected the physical and mental health of people around the world. This may be particularly true for patients with life-threatening diseases. We analyzed the level of fear of COVID-19 (FCV-19S), the prevalence of anxiety (HADS-A) and depression (HADS-D) in pulmonary arterial and chronic thromboembolic pulmonary hypertension (PAH and CTEPH) patients during the COVID-19 pandemic. In this multicenter prospective study, 223 patients (63% females, 66% PAH) with age range 18–90 years were included. The fear of COVID-19 was high, at a mean level of 18.9 ± 7.4 points. Anxiety (HADS-A ≥ 8 points) was diagnosed in 32% of all patients, depression (HADS-D ≥ 8 points) in 21%, and anxiety or depression in 38%. FCV-19S was higher in woman and in elderly people (*p* = 0.02; *p* = 0.02, respectively). In the multivariate analysis, FCV-19S higher than the median increased the odds ratio of anxiety, but not of depression (R 6.4 (95%CI 2.0–20.0), *p* = 0.002; OR 1.9 (0.9–3.9), *p* = 0.06, respectively). History of COVID-19 increased risk of both HADS-A and HADS-D. Patients with PAH and CTEPH, especially woman over 65 years and those who had been infected with COVID-19, may need additional psychological support due to fear of COVID-19, anxiety or depression.

## 1. Introduction

The COVID-19 pandemic has modified our lives and completely changed social functioning and healthcare systems’ organization [1]. The impact has been especially felt by patients with chronic diseases who require frequent contacts with the health service providers [1,2]. Patients suffering from chronic cardiac or pulmonary diseases have been advised to minimize medical contacts to reduce risk of COVID-19 infection, thus affecting both the level of care and patients’ feeling of security [3].

Pulmonary arterial hypertension (PAH) is a rare disease [4] characterized by pre-capillary pulmonary hypertension, which is defined by mean pulmonary arterial pressure (mPAP) ≥ 25 mmHg in right heart catheterization, elevated pulmonary vascular resistance (PVR) ≥ 3 Wood units, and normal pulmonary artery wedge pressure (PAWP) ≤ 15 mmHg, in the absence of other causes of pre-capillary pulmonary hypertension [5]. Chronic thromboembolic pulmonary hypertension (CTEPH) is a rare disease [6] characterized by pre-capillary pulmonary hypertension due to obstruction and remodeling of the pulmonary artery by major vessel thromboembolism [5]. Untreated PAH and CTEPH lead to progressive right heart failure and death [5]. Patients with PAH should be actively treated with specific drugs; CTEPH patients should be treated by means of pulmonary endarterectomy or pulmonary balloon angioplasty or/and specific drugs [7,8]. All PAH and CTEPH patients require regular assessment at expert pulmonary hypertension (PH) centers every 3–6 months to prevent PH deterioration [5].

The cumulative incidence of COVID-19 infection recognized in patients with PAH/CTEPH was similar to that in the general population, but outcomes can be worse, with the mortality rate around 12% in the United States [9] and similarly high in Europe [10]. The SARS-CoV-2 pandemic increased levels of stress, anxiety, insomnia, depression, and fear in the general population [11], but there are few data about these symptoms in PAH/CTEPH patients [12,13,14]. A tool proposed by Ahorsu et al. to measure the severity of stress response to the COVID-19 pandemic is the Fear of COVID-19 scale. Other scales are used to measure anxiety and/or depression [15,16].

The prevalence of anxiety and depression in PAH/CTEPH patients before the pandemic, which was measured using the Hospital Anxiety and Depression Scale, was high. Prevalence of anxiety was about 20.5–51% of patients [17,18]. Prevalence of depression was found in about 7.5–53% of patients [17,18].

This paper presents the report of a multicenter cross-sectional study performed in Poland. The aim of our study was to assess the prevalence of anxiety and depression, and the level and impact of fear of COVID-19 on anxiety and depression, in PAH and CTEPH patients during the third wave of the SARS-CoV-2 pandemic. Moreover, we identified clinical factors associated with higher levels of fear, anxiety and depression in PH patients.

## 2. Materials and Methods

### 2.1. Study Group

This prospective observational non-interventional study was performed in three pulmonary hypertension centers in Poland during the third wave of the COVID-19 pandemic. Consecutive patients with PAH and CTEPH were enrolled in the study between 15 April 2021 and 30 May 2021. All patients were 18 years old or older and had diagnosis of PAH or CTEPH confirmed by right heart catheterization and additional necessary compulsory tests according to the current guidelines [5]. Patients with CTEPH had distal disease or persistent pulmonary hypertension, despite pulmonary endarterectomy and balloon pulmonary angioplasty. The study protocol was approved by the Bioethics Committee of the Centre of Postgraduate Medical Education in accordance with the Declaration of Helsinki (number KBE 23/2021, date of approval: 14 April 2021). Written informed consent was obtained from all participants.

### 2.2. Methods

Patients who came for a routine visit to the three PH centers used for the study were asked to fill in two questionnaires: the Fear of COVID-19 scale (FCV-19S) and the Hospital Anxiety and Depression Scale (HADS) [15,19]. We chose FCV-19S and HADS tools to assess the mental status of PH patients for easier comparison of the obtained results because both scales are widely used in the clinics. FCV-19S is translated into various languages and validated in various countries [15,20,21,22,23,24,25]. Both scales have been translated into the Polish language [26,27] and validated for the Polish population.

The FCV-19S had good internal consistency (Cronbach’s alfa was 0.89 and 0.85) [25,27]. It is a psychometric tool that consists of 7 items. Answers were given on a scale ranging from 1 (strongly disagree) to 5 (strongly agree). Each patient chose a point from 1 to 5 for each statement and could get a total score of 7 to 35 points [15]. The higher the points, the higher the fear of COVID-19.

The Hospital Anxiety and Depression Scale reflects the general level of anxiety and depression. It consists of 16 items divided into anxiety (7 questions), depression (7 questions), and ratty (2 questions). Each item has four possible answers, and 0 to 21 points can be obtained for each subscale of anxiety or depression [16]. The same cut-off value of 8 or more in the HADS anxiety part (HADS-A) or HADS depression part (HADS-D) can be used to determine patients who are anxious or depressed [19,27]. A cut-off value of 11 or more in the HADS-A or HADS-D part is used to determine patients with severe anxiety or depression [27].

Medical records were reviewed to obtain information about the demographic characteristics of the patients, their clinical conditions and the treatment they had received.

### 2.3. Statistical Analysis

Statistical analysis was performed using Statistica software (by TIBCO Software Inc., license acquired from local distributor StatSoft Polska, Krakow, Poland), version 13.3. Categorical variables were presented as numbers and percentages, while continuous variables were presented as medians and interquartile ranges or means and standard deviations. Data distribution was tested using the Kolmogorov-Smirnov test. For group comparisons, Chi-square, Fisher’s exact-test, paired t-test or the Mann-Whitney U were used as appropriate. A multivariate regression analysis was performed to identify factors associated with fear of COVID-19, HADS-A and HADS-D. Statistical differences was considered significant for *p* ≤ 0.05.

## 3. Results

### 3.1. Study Group

**Figure 1:**
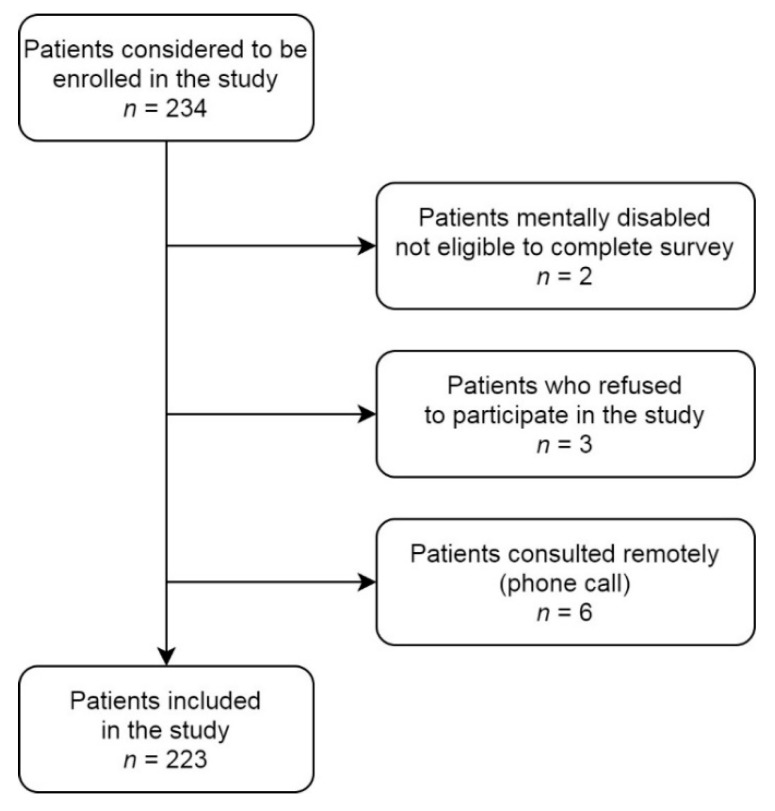
Patients’ disposition participate in the study.

A total of 234 consecutive patients were screened as potential study participants. Two were excluded from the study due to mental illness which made it impossible to complete the questionnaires and sign the informal consent, three did not agree to participate in the study, and six were having teleconsultation at that time. Figure 1 shows patients’ disposition to participate in the study.

A total of 223 patients were finally included in the study. Most of the patients were female (141, 63%). The patients’ median age was 59 years (range 18–90). In the study group, 147 (66%) patients suffered from PAH, while 76 (44%) patients had CTEPH. There were more female patients in the PAH group (108, 73%), whereas there were more men in the CTEPH group (43, 56%; *p* < 0.001). Both PAH and CTEPH groups differed in age, with the PAH patients generally younger (median age 56 (18–88) vs. 66 (22–90), *p* = 0.002). The PAH patients had a longer history of PH disease than CTEPH patients (8.6 ± 8.1 years vs. 5.0 ± 4.5 years; *p* = 0.001) and more impaired functional WHO class (2.5 ± 0.6 vs. 2.1 ± 0.8; *p* = 0.002). As many as 17% of patients had a history of COVID-19. Only 43% of patients had been vaccinated against COVID-19, with no differences between both groups in this regard. The most common reason to refuse COVID-19 vaccines among PH patients was fear of side effects. The characteristics of the study group are presented in Table 1.

### 3.2. Fear of COVID-19 and Hospital Anxiety and Depression

Table 2 shows the manifestation of fear of COVID-19, anxiety and depression in the patients.

The median (IQ) of fear of COVID-19 scale was 19 (13–24) for both patient groups. The median (IQ) of HADS-A was 6 (3–9) and the median (IQ) of HADS-D was 4 (1–7). There were no significant differences in FCV-19S and in the frequency of anxiety and depression between the PAH and CTEPH groups.

**Figure 2:**
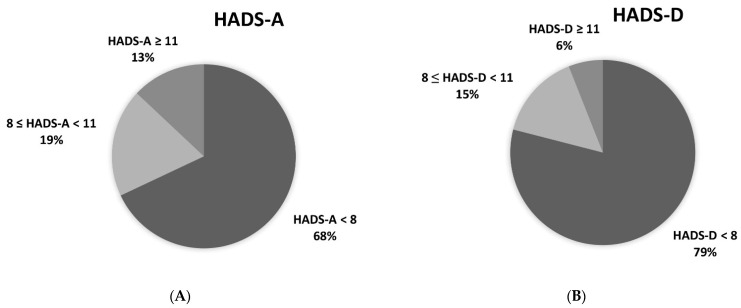
Hospital anxiety and depression scale results (HADS) in PAH and CTEPH patients; (**A**) HADS-A (anxiety subscale): HADS-A < 8 normal value, HADS-A ≥ 8 < 11 moderate anxiety (anxiety suspected), HADSA ≥ 11 severe anxiety (anxiety probable); (**B**) HADS-D (depression subscale): HADS-D < 8 normal value, HADS-D ≥ 8 < 11 moderate depression (depression suspected), HADSA ≥11 severe depression (depression probable).

A detailed distribution of HADS scores is shown in Figure 2. Patients revealed elevated HADS score for anxiety in 32% and for depression in 21% and anxiety or depression in 38%.

**Figure 3:**
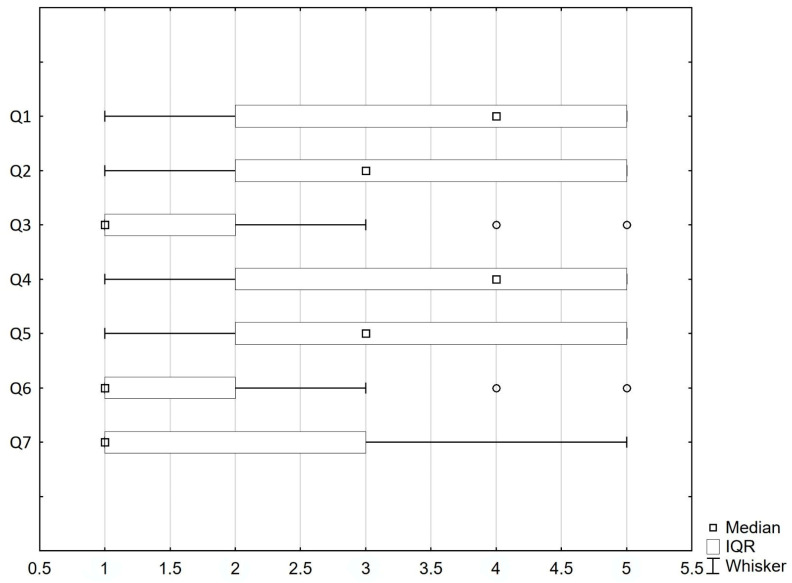
Fear of COVID-19 scale: Individual item results (Q1–Q7), median, IQR. Q1—I am most afraid of coronavirus-19; Q2—It makes me uncomfortable to think about coronavirus-19; Q3—My hands become clammy when I think about coronavirus-19; Q4—I am afraid of losing my life because of coronavirus-19; Q5—When viewing news and stories about coronavirus-19 on social media, I become nervous or anxious; Q6—I cannot sleep because I’m worried about getting coronavirus-19; Q7—My heart races or palpitates when I think about getting coronavirus-19. Point 1—I strongly disagree, point 5—I strongly agree.

The majority of patients experienced fear of COVID-19—the median (IQ) on the fear of COVID-19 scale was 19 (13–24). Most of the patients reported that they were afraid of contracting COVID-19 and losing their life as a result. Unconditional physical stress responses to the COVID-19 pandemic, such as sweating hands, palpitations and insomnia, were reported much less frequently. A detailed distribution of answers to the seven questions in the Fear of COVID-19 Scale is presented in Figure 3.

Results of the fear of COVID-19 scale, HADS-A and HADS-D, stratified by clinical data, are presented in Table 3. The FCV-19S was significantly higher in women than in men (0 (15–25) vs. 17 (12–23), *p* < 0.05) and in older than younger patients (2 (15–27) vs. 18 (13–22), *p* < 0.05). There was no significant difference in FCV-19S between PAH and CTEPH patients, between subgroups of PAH, and between patients in different WHO functional classes; FCV-19S was also found to be independent of duration of the disease, history of COVID-19, and history of vaccination against COVID-19.

The level of anxiety (HADS-A) was higher in women than in men ( (3–9) vs. 5 (2–8), *p* < 0.05), and in patients with a history of COVID-19 (8 (5–10) vs. 5 (3–8), *p* < 0.05).

The level of depression (HADS-D) was higher in older than younger patients (5 (3–8) vs. 3 (1–7), *p* < 0.05), in patients in the WHO functional class 3 or 4 than those in class 1 or 2 (5 (3–9) vs. 3 (1–6), *p* < 0.001), and in patients with a history of COVID-19 (7 (2–9) vs.3 (1–7), *p* < 0.05).

Fear of COVID had an impact on patient anxiety, which was measured by HADS-A. In the univariate regression logistic analysis, FCV-19S higher than the median level and history of COVID-19 increased the odds ratio of anxiety in the HADS scale. In the multivariate regression analysis, the same parameters—FCV-19S higher than median and history of COVID-19 disease—increased the odds ratio of HADS-A (OR 6.4 95%CI (2.0–20.0), *p* = 0.002; OR 3.5 95%CI (1.5–7.7), *p* = 0.02, respectively). Results are presented in Table 4.

Fear of COVID-19 had a low impact on depression, which was measured by HADS-D in the patients. In the univariate logistic analysis, FCV-19S higher than the median level, history of COVID-19, and WHO functional class 3 or 4 increased the odds ratio of depression in the HADS scale. In the multivariate analysis, history of COVID-19 and WHO functional class 3 or 4 increased the odds ratio of depression in HADS-D (OR 3.2 (1.4–7.0), *p* = 0.004; OR 2.4 (1.2–4.8), *p* = 0.01, respectively). Results are presented in Table 5.

## 4. Discussion

Data on fear, anxiety and depression among PH patients during the COVID-19 pandemic are scarce. The COVID-19 pandemic and its associated social isolation have worsened the mental state of people and increased anxiety and depression in the general population [11,28]. It is known that the mortality rate in PAH and CTEPH patients compared to the general population is high [9,10]. Moreover, the pandemic has made it difficult to access specialized health care [13]. Interruption of care could have negative consequences for the health of PH patients [2,13,29]. All these factors can increase their level of anxiety and depression.

A phone visit or a video-chat visit is an important and safe method of doctor–patient contact, reducing the risk of transmission of COVID-19 infection. At the end of the third wave of the pandemic in Poland, when the incidence of COVID-19 decreased significantly, we aimed to assess the patients to ensure that treatment of PAH was effective before COVID-19 infections will rise again.

The FCV-19S is a psychometric tool created during the beginning of the SARS-COV2 pandemic to assess emotional response to the pandemic [15]. The FCV-19S may reflect depression, anxiety, stress, mental well-being, generalized anxiety disorder, psychological distress, post-traumatic disorder, and specific phobia [15,20,22,23,25]. The mean level of FCV-19S in our study patients was 18.9 ± 7.4 points (median 19 (13–24)). Some studies on general populations in various countries found higher or lower fear of COVID-19, with mean FCV-19S from 13.2 to 27.3 points [15,20,22,23,24,25]. Comparison of the results is difficult due to the different times of assessment, different ages of the examined populations, and other varying characteristics. The higher the FCV-19S points, the higher the level of fear and anxiety due to the COVID-19 pandemic. The fear of COVID-19 in PH patients is higher than in the general population in Poland. The FCV-19S was measured in the general population by internet survey one year before our study, from 15 May 2020 to 15 June 2020. The mean FCV-19S in the general population was 13.16 ± 4.8 [25]. Interestingly, the level of fear of COVID-19 in PH patients is similar to the FCV-19S in other life-treating populations, namely cancer patients. According to data from a prospective multicenter study, the impact of COVID-19 on the anxiety level of cancer patients in Poland was 18.5 ± 7.4 [30]. The described study was conducted a year earlier than our study, from 11 May to 15 May 2020, which was two months after the pandemic began. We noticed that, although the pandemic had lasted more than one year and vaccinations against COVID-19 were available, the level of fear of COVID-19 in PH patients still remained high.

In our study, fear of COVID-19 was higher in female patients. This result is consistent with those of previous studies [20,22,24,25,31]. In our study, FCV-19S positively correlated with age. This is in concordance with results from another study [25], but disagrees with others where FCV-19S was higher in younger patients [30] or age had no impact on FCV-19 [23].

We used the HADS scale to identify anxiety and depression. Meta-analysis proved that the HADS scale is good for the detection and evaluation of worsening symptoms of anxiety disorders and depression in general populations and in different groups of patients, such as somatic, psychiatric and primary care patients [32].

Around 32% of PAH/CTEPH patients in our study had anxiety, and 21% had depression. Our results are similar to those of a study on mental disorder in PAH patients during the SARS-CoV-2 pandemic. Park et al. measured the level of anxiety and depression using the HADS scale during the first wave of the COVID-19 pandemic. Among 152 PAH patients in this study by Park et al., the prevalence of anxiety in HADS-A was 34% at baseline and the prevalence of depression in HADS-D was 23% at baseline. The prevalence of mental disorders did not change after 232 days of observation, and was 33% for anxiety (*p* = 0.07) and 23% for depression (*p* = 0.13%) [12]. Moreover, the authors examined the quality of life of the patients but observed no changes between the baseline quality and the quality during follow-up [12]. They hypothesized that the pandemic did not significantly affect mental disorders and the quality of life of PAH patients. However, in this study PAH patients were not examined before and during the COVID-19 pandemic, but were examined twice during the pandemic. Our study was performed during the third wave of the COVID-19 pandemic.

In studies conducted before the SARS-CoV-2 pandemic, the prevalence of anxiety and depression in PH patients was high, with 19–51% for anxiety and 7.5–56% for depression [17,18,33,34,35,36,37,38]. Somaini et al. used the HADS and reported significant differences in the prevalence of anxiety and depression between incident and prevalent PAH/CTEPH patients [17]. The prevalence of anxiety was 51% in incident PH patients and 24% in prevalent PH patients. The prevalence of depression was 53% in incident PH patients and 21% in prevalent PH patients. Mental disturbances improved during PH therapy [17]. In our study, only five patients had incident PH, and our results are similar to those of the prevalent PH patients in the study of Somaini et al.

We found no difference in depression levels between PAH and CTEPH patients. This result disagrees with that of a retrospective observational study by Pfeuffer et al., who reported that depression was more frequent in CTEPH than in PAH patients (56% vs. 30%; *p* = 0.03) using the HADS scale. Similarly, they, and another author, found no difference in anxiety levels between CTEPH and PAH patients [34,38]. We observed a tendency to higher prevalence of depression and anxiety in CTEPH patients than in PAH patients but the difference was not significant.

The level of anxiety and depression in our patients was probably high because of many triggers, including anxiety about the prognosis of the disease and worries about effectiveness of treatment. Other triggers could be associated with the COVID-19 pandemic, such as fear of aggravation of the disease due to COVID-19 infection, anxiety regarding delayed contact with healthcare providers due to the COVID-19 pandemic, and awareness of treatment interruption due to lack of PH drugs. Depression and anxiety correlate with impairment of different aspects of the quality of life of PH patients [17,18,33,37,38,39] and could worsen prognosis [39]. Moreover, the COVID-19 pandemic was reported to have elevated the anxiety of parents of children with pulmonary arterial hypertension, as 34.5% of parents revealed very high levels of anxiety measured by the general anxiety disorder (GAD-7) [14].

Before the COVID-19 pandemic, anxiety and depression were diagnosed using the HADS scale in 11% and 10% of a general elderly population, respectively [40]. During pandemic isolation in China, 68% of the general population suffered from anxiety disorders [11]. During the second wave of COVID-19 in Poland, around 59.2% of the total population had mental disorders [28]. In our study, 38% of patients had anxiety or depression, which was less than the percentage of the general population with anxiety or depression during the pandemic and the percentage of incident PH patients with anxiety or depression before the pandemic. We speculate that patients with PH had a high level of anxiety and depression due to the rare life-threatening disease, and the additional impact of fear of COVID-19 increased the anxiety and depression, but not as much as in the general population. Our thesis is confirmed by the result of the multivariate regression analysis which showed that FCV-19S higher than the median caused a greater odds ratio for anxiety but not for depression. On the other hand, the WHO functional class 3 or 4, which reflects a high severity of the PH disease, increased the odds ratio of depression in the multivariate analysis. The same situation was observed in patients suffering from cancer and other life-threatening conditions. In cancer patients, the FCV-19S and anxiety related to COVID-19 were significantly lower than the cancer-associated anxiety [30]. This might be because patients were probably more afraid of the consequences of their serious disease than the risk of COVID-19 infection [30]. This situation changed when patients suffered COVID-19 infection. When alive, they had high odds ratio to anxiety and depression.

By 31 May 2021, there were 2,875,136 confirmed cases of COVID-19 in Poland, with 74,152 deaths reported [41]. This means that 7.5% of Polish people have suffered from COVID-19 infection, with 2.4% of those infected dying from the disease. In our study, 17% of patients had suffered from COVID-19 infection. The incidence was even greater, because we excluded from the study five patients who died from COVID-19 and one whose enrolment was considered but not included in the study (Figure 1). Patients who had a history of COVID-19 infection had greater anxiety and depression but not fear of COVID-19. This might be because fear is not constant over time and fear of COVID-19 is most likely to measure the relatively short-term stress response.

Current guidelines for anxiety and depression management in the general population recommend psychotherapy as a first step in reducing anxiety and a combination of drugs and psychotherapy in reducing depression [42]. Such interventions should also be an important part of palliative care in patients with pulmonary hypertension [43]. In our study, only 35% of patients diagnosed with anxiety and depression knew about their mental problems and were receiving drugs or/and psychotherapy. Treatment of depression had no impact on the odds ratio of anxiety or depression on the HADS scale and had no impact on fear of COVID-19.

Despite the fact that significant advances have been made in the diagnosis and treatment of PH in recent years, it continues to have a chronic progressive course that worsens the prognosis. PH is a life-threatening disease that affects various aspects of a patient’s and their carer’s life [44]. PAH and CTEPH often require reorganization of the patient’s entire life [45]. PAH affects physical activities, travel, and social opportunities. Patients with PH feel socially isolated due to lack of knowledge and understanding among relatives and the public. Pulmonary hypertension reduces the possibility of paid work and employment for both patients and their relatives, which is of great importance for their finances [45]. All of these circumstances may increase anxiety and depression regardless of the COVID-19 pandemic.

Our study has some limitations. The study group was relatively small. The time from completion of the first questionnaire to the last was long, taking one and a half months. It is known that the level of anxiety is not constant over time. The long time taken to complete the entire questionnaires resulted from the patients’ visiting the PH centers every 2–4 months. The patients filled in the questionnaires personally and signed a written consent during those periodical visits to limit unnecessary travel and, thus, exposure to COVID-19. Moreover, we had no data on the level of anxiety and depression in the study group before the COVID-19 pandemic, so we could only refer to other PH populations. In the future, we plan to re-assess anxiety and depression in the same study group after the COVID-19 pandemic. The level of anxiety might have varied at different time points in the pandemic. We conducted our study during the third wave of the pandemic, more than a year after the pandemic began, when the adaptation process was already advanced and may have affected the level of anxiety and depression in the examined group. Furthermore, we did not collect other general data that could have an impact on the obtained anxiety or fear results, such as education, place of residence, marital status or media habits.

## 5. Conclusions

Pulmonary hypertension is a disease with a significant impact on the psychological, emotional and social functioning of patients and their families. Psychological and social support is recommended by current guidelines. During the COVID-19 pandemic, when the organization of health care has changed and the level of fear of COVID-19 in PH patients is high and may impact their mental wellbeing, active screening for anxiety and depression in PH patients is crucial. Fear of COVID-19 has an impact on anxiety but not on depression. PH patients, especially women, aged over 65 years, in an advanced functional WHO class, and who had a history of COVID-19 infection may need psychological or pharmacological support due to anxiety or depression. Further studies on the impact of the ongoing COVID-19 pandemic on mental wellbeing in PH patients are needed. A better understanding of the relationship between fear of COVID-19, anxiety and depression will help improve the care of patients with PH, their quality of life, and patient–doctor cooperation.

## Figures and Tables

**Table 1:** Characteristics of the study group patients according to the pulmonary hypertension type.

	Total Study Group *n* (%) or Mean (SD)	PAH *n* (%) or Mean (SD)	CTEPH *n* (%) or Mean (SD)	*p* < 0.05 PAH vs. CTEPH
Number of patients	223 (100%)	147 (66%)	76 (34%)	
Females/males	141/82 (63%/37%)	108/39 (73%/27%)	33/43 (43%/56%)	0.00001 *
Age, years	59 (18–90)	56 (18–88)	66 (22–90)	0.002 *
Duration of disease, years	7.4 ± 7.3	8.6 ± 8.1	5.0 ± 4.5	0.001 *
PAH patients				
Idiopathic PAH		75 (51%)		
Heritable PAH		5 (3%)		
PAH associated with CHD		34 (23%)		
PAH associated with CTD		27 (18%)		
PAH porto-pulmonary		5 (3%)		
Drug-induced PAH		1 (0.6%)		
PAH monotherapy		35 (24%)		
PAH two drugs		58 (40%)		
PAH three drugs		54 (36%)		
CTEPH-BPA			58 (76%)	
CTEPH-PEA			17 (22%)	
CTEPH monotherapy (riociguat or sildenafil)			59 (78%)	
WHO functional class	2.4 ± 0.7	2.5 ± 0.6	2.1 ± 0.8	0.002 *
1	17 (8%)	3 (2%)	14 (18%)	
2	109 (49%)	73 (50%)	36 (47%)	
3	85 (38%)	61 (41%)	24 (32%)	
4	12 (5%)	10 (7%)	2 (3%)	
Vaccinated against COVID-19	96 (43%)	56 (38%)	40 (53%)	0.03 *
COVID-19 disease	37 (17%)	21 (14%)	16 (21%)	0.19
History of depression	29 (13%)	22 (15%)	7 (9%)	0.23
Concomitant disease	142 (58%)	85 (58%)	57 (75%)	0.01 *
Arterial hypertension	100 (41%)	60 (41%)	40 (53%)	0.10
Diabetes	37 (15%)	25 (17%)	12 (16%)	0.81
COPD	20 (8%)	11 (7%)	9 (12%)	0.28
Coronary artery disease	32 (13%)	22 (15%)	10 (13%)	0.72
Neoplasm	21 (9%)	11 (7%)	10 (13%)	0.17
Obesity, BMI ≥ 30 kg/m^2^	59 (24%)	36 (24%)	23 (30%)	0.35

PH—Pulmonary hypertension, PAH—Pulmonary arterial hypertension, PAH-CHD—Pulmonary arterial hypertension related to congenital heart disease, IPAH—Idiopathic pulmonary hypertension, PAH-CTD—Pulmonary arterial hypertension associated with connective tissue disease, PAH-porto-pulmonary—Pulmonary arterial hypertension associated with portal hypertension CTEPH—Chronic thromboembolic pulmonary hypertension, BPA—Balloon pulmonary angioplasty, PEA—Pulmonary endarterectomy, COPD—Chronic obstructive pulmonary disease, WHO—World Health Organization, *p* < 0.05 *.

**Table 2:** Median and mean scores in FCV-19S, HADS-A, HADS-D, and HADS-R in the general study group.

	All Patients *n* (%); Median (IQR) or Mean (SD) *n* = 223	PAH *n* (%); Median (IQR) or Mean (SD) *n* = 147	CTEPH *n* (%); Median (IQR) or Mean (SD) *n* = 76	*p*-Value
Fear of COVID-19, points	19 (13–24) 18.9 ± 7.4	20 (14–25) 19.3 ± 7.4	17 (12.5–23) 17.9 ± 7.3	0.12
HADS-A, points	6.0 (3–9) 6.0 ± 3.7	6 (3–9) 6.1 ± 3.6	6 (2.5–9) 5.8 ± 3.8	0.54
HADS-D, points	4 (1–7) 4.5 ± 3.6	3.0 (1–7) 4.3 ± 3.5	4 (1–7) 4.8 ± 3.8	0.49
HADS ratty, points	2 (1–4) 2.7 ± 1.8	2 (1–4) 2.7 ± 1.8	2 (1–4) 2.7 ± 2.0	0.84
Patients with HADS-A ≥ 8 points	71 (32%)	45 (31%)	26 (34%)	0.58
Patients with HADS-A ≥ 11 points	29 (13%)	22 (15%)	22 (15%)	0.22
Patients with HADS-D ≥ 8 points	46 (21%)	28 (19%)	18 (24%)	0.41
Patients with HADS-D ≥ 11 points	14 (6%)	8 (5%)	6 (8%)	0.47
Patients with HADS-A ≥ 8 points or HADS-D ≥ 8 points	84 (38%)	53 (36%)	31 (41%)	0.56

FCV-19S—Fear of COVID-19 Scale, HADS—Hospital anxiety and depression scale, HADS-A—Hospital anxiety and depression scale-anxiety subscale, HADS-D—Hospital anxiety and depression scale-depression subscale; HADS ratty—Hospital anxiety and depression scale-ratty subscale; PAH—Pulmonary arterial hypertension, CTEPH—Chronic thromboembolic pulmonary hypertension.

**Table 3:** Comparison of the level of fear of COVID-19, anxiety (HADS-A) and depression (HADS-D) in the study group.

	FCV-19S Median (IQR)	*p*-Value	HADS-A Median (IQR)	*p*-Value	HADS-D Median (IQR)	*p*-Value
PH type		0.12		0.54		0.49
All types of PAH	19 (13–24) 18.9 ± 7.4		6 (3–9) 6.0 ± 3.7		4 (1–7) 4.5 ± 3.6	
IPAH	20 (12–27)		5 (3–9)		3 (1–7)	
PAH-CHD	18 (15–23)		7 (4–9)		3.5 (1–7)	
PAH-CTD	20 (14–27)		7 (4–9)		5 (3–8)	
PAH-porto-pulmonary	23 (17–24)		6 (4–7)		1 (1–2)	
Heritable PAH	21 (18–21)		6 (5–7)		1 (1–4)	
CTEPH	17 (12.5–23)		6 (2.5–9)		4 (1–7)	
Gender		0.024 *		0.026 *		0.42
female	20 (15–25)		6 (3–9)		4 (1–7)	
male	17 (12–23)		5 (2–8)		3 (1–7)	
Age		0.023 *		0.48		0.003 **
<65 years	18 (13–22)		6 (3–9)		3 (1–7)	
≥65 years	22 (15–27)		5 (3–9)		5 (3–8)	
WHO functional class		0.15		0.09		<0.001 ***
1–2	17.5 (13–23)		5 (3–8)		3 (1–6)	
3–4	20 (14–25)		6 (3–10)		5 (3–9)	
History of COVID-19		0.84		0.006 **		0.003 **
yes	19 (13–23)		8 (5–10)		7 (2–9)	
no	18.5 (13–24)		5 (3–8)		3 (1–7)	
Vaccination against COVID-19		0.16		0.27		0.86
yes	19.5 (14–25.5)		5 (2–9)		3.5 (1–7)	
no	18 (12–23)		6 (3–8)		4 (1–7)	

PH—Pulmonary hypertension, PAH—Pulmonary arterial hypertension, PAH-CHD—Pulmonary arterial hypertension related to congenital heart disease, IPAH—Idiopathic pulmonary arterial hypertension, PAH-CTD—Pulmonary arterial hypertension associated with connective tissue disease, PAH-associated with portal hypertension, CTEPH—Chronic thromboembolic pulmonary hypertension, WHO—World Health Organization, FCV-19S—Fear of COVID-19 Scale; HADS-A—Anxiety part of the hospital anxiety and depression scale, HADS-D—Depression part of the hospital anxiety and depression scale *p* < 0.05 *, *p* < 0.01 **, *p* < 0.001 ***.

**Table 4:** Impact of different factors on anxiety in the HADS scale (HADS-A ≥ 8): Results of the univariate and multivariate logistic regression analysis.

	Univariate Analysis	*p*-Value	Multivariate Analysis	*p*-Value
	HADS-A ≥ 8 OR (95%CI)		HADS-A ≥ 8 OR (95%CI)	
FCV-19S ≥ median	5 (2.6–9.4)	0.0000 *	6.4 (2–20)	0.002 *
History of COVID-19	3.1 (1.5–6.4)	0.002 *	3.5 (1.5–7.7)	0.02 *
Vaccination against COVID-19	1.1 (0.6–2)	0.67		
WHO functional class 3–4	1.7 (0.9–2.9)	0.07		
History of depression (drugs or psychotherapy)	1.9 (0.8–4.2)	0.11		
Age ≥ 65 years	1.1 (0.6–1.9)	0.78		
Female gender	1.4 (0.8–2.6)	0.22		

FCV-19S—Fear of COVID-19 Scale, HADS—Hospital anxiety and depression scale, HADS-A—Anxiety part of the hospital anxiety and depression scale, * *p* < 0.05.

**Table 5:** Impact of different factors on depression in the HADS scale (HADS-D ≥ 8): results of the univariate and multivariate regression analysis.

	Univariate Analysis	*p*-Value	Multivariate Analysis	*p*-Value
	HADS-D ≥ 8 OR (95%CI)		HADS-D ≥ 8 OR (95%CI)	
FCV-19S ≥ median	2.1 (1–4.2)	0.02 *	1.9 (0.9–3.9)	0.06
History of COVID-19	3.4 (1.6–7.3)	0.002 *	3.2 (1.4–7)	0.004 *
Vaccination against COVID-19	1 (0.5–1.9)	0.9		
WHO functional class 3–4	2.7 (1.4–5.3)	0.003 *	2.4 (1.2–4.8)	0.01 *
History of depression (drugs or psychotherapy)	1.6 (0.6–3.8)	0.32		
Age ≥ 65 years	1.6 (0.8–3.1)	0.1		
Female gender	0.7 (0.2–2.2)	0.6		

FCV-19S—Fear of COVID-19 Scale, HADS—Hospital anxiety and depression scale, HADS-D—Depression part of the hospital anxiety and depression scale, * *p* < 0.05.

## Data Availability

Data sharing is not applicable to this article.
